# Towards Enhancing Traffic Sign Recognition through Sliding Windows

**DOI:** 10.3390/s22072683

**Published:** 2022-03-31

**Authors:** Muhammad Atif, Tommaso Zoppi, Mohamad Gharib, Andrea Bondavalli

**Affiliations:** 1 Department of Mathematics and Informatics, 50142 Florence, Italy; muhammad.atif@unifi.it (M.A.); tommaso.zoppi@unifi.it (T.Z.); 2 Institute of Computer Science, University of Tartu, 51009 Tartu, Estonia; mohamad.gharib@ut.ee

**Keywords:** traffic sign recognition, sliding windows, meta learning, deep learning, classification

## Abstract

Automatic Traffic Sign Detection and Recognition (TSDR) provides drivers with critical information on traffic signs, and it constitutes an enabling condition for autonomous driving. Misclassifying even a single sign may constitute a severe hazard, which negatively impacts the environment, infrastructures, and human lives. Therefore, a reliable TSDR mechanism is essential to attain a safe circulation of road vehicles. Traffic Sign Recognition (TSR) techniques that use Machine Learning (ML) algorithms have been proposed, but no agreement on a preferred ML algorithm nor perfect classification capabilities were always achieved by any existing solutions. Consequently, our study employs ML-based classifiers to build a TSR system that analyzes a sliding window of frames sampled by sensors on a vehicle. Such TSR processes the most recent frame and past frames sampled by sensors through (i) Long Short-Term Memory (LSTM) networks and (ii) Stacking Meta-Learners, which allow for efficiently combining base-learning classification episodes into a unified and improved meta-level classification. Experimental results by using publicly available datasets show that Stacking Meta-Learners dramatically reduce misclassifications of signs and achieved perfect classification on all three considered datasets. This shows the potential of our novel approach based on sliding windows to be used as an efficient solution for TSR.

## 1. Introduction

Intelligent transportation systems are nowadays of utmost interest for researchers and practitioners as they aim at providing advanced and automatized functionalities, such as obstacle detection, traffic sign recognition, car plate recognition, and automatic incident detection or stopped vehicle detection systems. Particularly, Traffic Sign Detection and Recognition (TSDR) systems aim at detecting (TSD) and recognizing (TSR) traffic signs from images or frames sampled by sensors [1,2,3] installed on vehicles (e.g., webcams). Those systems synergize with the human driver, who may misinterpret or miss an important traffic sign, potentially leading to accidents that may generate safety-related hazards [4]. When integrated into intelligent vehicles [5,6], in terms of Advanced Driver-Assistance Systems (ADAS) [2,7,8,9], TSDR can automatically provide drivers with actionable warnings or even trigger reaction strategies (e.g., automatic reduction of speed, braking) that may be crucial to avoid or reduce the likelihood of accidents [3,10].

Humans are expected to naturally miss or misinterpret a traffic sign occasionally because of being distracted [11]. Similarly, to humans, TSDR systems are also subject to error as they may misinterpret or miss a traffic sign. This could happen due to various reasons, such as unsatisfactory road situations, imperfect traffic sign state, adverse environmental conditions (e.g., foggy weather [12]) or imperfect analysis processes. Nevertheless, researchers and practitioners are trying to minimize misclassifications at the automatic TSDR side, which is expected to increase safety by providing drivers with accurate and timely notifications.

Available TSD systems can precisely extract areas of an image or a frame, which are supposed to contain a traffic sign. Thereto, TSR systems that embed Machine Learning (ML) algorithms [13,14,15,16], process features that are extracted from those images through feature descriptors (e.g., Histogram of Oriented Gradients (HOG) [17], Local Binary Pattern (LBP) [16] to recognize traffic sign categories [18,19]. Alternatively, deep learning algorithms, such as AlexNet, googLeNet [20] can directly process images coming from sensors and classify them according to internal representation learning processes, which are orchestrated through multiple convolutional and fully connected layers. Throughout the years, many studies tackled TSR [21,22,23] using different feature descriptors and ML-based classifiers. Different combinations of such classifiers and features have been proven to generate heterogeneous classification scores [15,19,24,25,26,27] motivating the need for comparisons to discover the optimal classifier for a given TSR problem [3,28,29].

Regardless of the outcomes of comparison studies, most of the existing solutions for TSR process a single image or frame and output a classification result. Instead, vehicles gradually approach traffic signs during their road trips, generating sequences of images: the closer the vehicle is to the traffic sign, the better the quality of the image, even under slightly different environmental conditions. Therefore, the problem of TSR naturally scales to knowledge extraction from a set or sequence of images that potentially contain traffic signs. Consequently, the classification process should not depend only on a single frame to make a decision; instead, it should build on the knowledge acquired as the vehicle moves forward, i.e., the sequence of images.

This study considers a sliding window of images to commit classification rather than classifying frames individually. First, we process each image with the most effective single-frame classifier for TSR: then, we combine classification scores assigned to images in the sliding window to provide a unified and improved classification result. Such a combination is performed by appropriate Meta-Learners [30], which suit model combination, and therefore, show potential to be applied in such a context.

We conduct an experimental evaluation by processing three public datasets, namely, (i) German Traffic Sign Recognition Benchmark (GTSRB) [31] (ii) BelgiumTSC [32], and (iii) the Dataset of Italian Traffic Signs (DITS) [33], which report on sequences or unordered sets of images of traffic signs. From each image, we extracted 12 different feature sets, which use handcrafted features (HOG [17], LBP [16]), deep features (from AlexNet [34], ResNet-18 [35]), and their combinations, to debate their impact in TSR. Those features were fed to supervised classifiers as Decision Trees [36], Random Forests [37], k-th Nearest Neighbour (K-NN, [13]), Linear Discriminant Analysis Classifier (LDA) [38], Support Vector Machines (SVMs) [14], and AdaBoost [39]. We also exercised single-frame classifiers that do not rely on feature descriptors as deep learners, namely Inceptionv3 [40], MobileNet-v2 [41] and AlexNet [34]. We used the classifiers above both as single-frame classifiers and as base-level learners of a Stacking meta-learner, which aggregates individual classification scores into sliding windows. The meta-level classifier for Stacking was experimentally chosen out of supervised (non-deep) classifiers, the Majority Voting [42] and Discrete Hidden Markov Model (DHMM, [43]).

Additionally, we compare the classification performance of those meta-learners with Long Short-Term Memory (LSTM) networks, which naturally deal with sequences of data coming at different time instants. We trained those LSTM networks on the same sliding windows of images processed through Stacking. Results show how single-frame classifiers achieve 100% accuracy on the GTRSB, 99.72% on BelgiumTSC and 96.03% on DITS datasets. Then, we applied our approach based on sliding windows by using LSTM networks and stacking meta-learners, finding that both approaches greatly improve the accuracy of TSR: particularly, specific stacking meta-learners achieved perfect accuracy (i.e., no misclassifications at all) on the three datasets by using a sliding window of two or three images.

Summarizing the contribution and novelty of the paper mainly lies in the following items:

- a deep literature review about ML-based TSR;
- presentation of an approach based on sliding windows of frames to be processed either by meta-learners or LSTM;
- an experimental campaign that relies on heterogeneous and public datasets of traffic signs; and finally
- a discussion of results that clearly shows how a sliding window of at least two items, deep base-level classifiers and K-NN as stacking meta-learner allow achieving perfect TSR on all datasets considered in the study, dramatically improving the state of the art.

The rest of the paper is organized as follows: Section 2 elaborates on related works and a review of existing TSR systems. Section 3 expands on our approach based on sliding windows. Section 4 reports on our experimental setup and methodology, classifiers, and feature sets to compare different TSR systems. Finally, Section 5 discusses and comments on those experimental results, letting Section 6 conclude the paper.

## 2. Background on Traffic Sign Recognition

### 2.1. Classifiers for TSR

In the last decade, researchers, practitioners, and companies devised automatic TSR systems to be integrated into ADAS. Amongst all the possible approaches, most TSR systems rely on the same main blocks, namely: (i) Dataset creation/identification, (ii) pre-processing (e.g., resizing, histogram equalization), (iii) Feature extraction and supervised model learning, or (iv) model learning through deep classifiers (i.e., deep learners).

**Figure 1:**
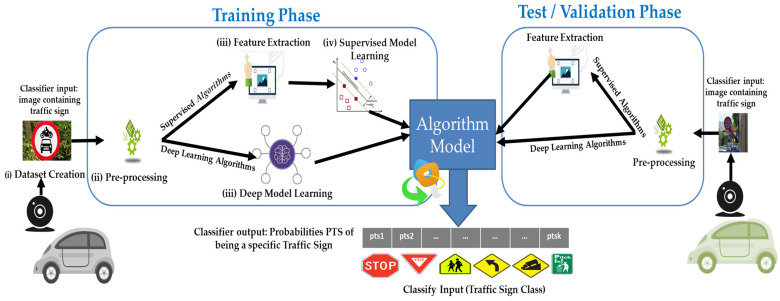
Block diagram of traffic sign recognition through deep learners or supervised classifiers.

As depicted in Figure 1, these building blocks interact with each other sequentially. Each image in the dataset is pre-processed to make feature extraction easier. These features are then fed into the classifier, either for training or for testing (right of Figure 1) if the model was already learned. Alternatively (see bottom left of Figure 1) we could rely on deep learning algorithms, which—unlike traditional supervised classifiers—embed representation learning, and therefore, do not require feature extraction.

Regardless of their type, classifiers output Probabilities of Traffic Sign categories (PTS), or rather, assign probabilities belonging to any known class of traffic signs to each image. The category of a traffic sign corresponds to the highest probability in PTS which defines the predicted class of a given image.

### 2.2. Related Works on Single-Frame TSR

Feature extractors and supervised classifiers have been arranged differently to minimize misclassifications in a wide variety of domains. Soni et al. [24] processed the Chinese traffic sign dataset through SVM, trained on the HOG or LBP after Principal Component Analysis (PCA), reaching an accuracy of 84.44%. A similar setup was used by Manisha and Liyanage [21], who achieved 98.6% accuracy on vehicles moving at 40–45 km/h. Moreover, Matoš et al. [22] used an SVM trained on HOG features and achieved recognition of 93.75% accuracy on the GTSRB dataset. The same dataset was used in [44], where Extreme Learning Machine (ELM) improved accuracy to 96%. Agrawal and Chaurasiya [45] extracted HOG features from the traffic signs of the GTSRB dataset and applied PCA for the dimensionality reduction to obtain an accuracy of 73.99%, 66.46%, 91.86% on denial, mandatory and danger traffic sign categories. Similar studies as [15,46,47] processed the same datasets with different feature sets and algorithms, obtaining similar scores.

Deep learners and the Viola–Jones framework allowed the authors of [8] to enhance classification on the GTSRB dataset with up to 90% of accuracy. Li et al. [28] proposed a new Convolutional Neural Network and trained on the GTSRB and BelgiumTSC datasets. The proposed architecture achieved an accuracy of 98.1% and 97.4% on BelgiumTSC and GTSRB datasets, respectively. In [48], the fifteen-layer WAF-LeNet network reached a detection accuracy of 96.5% on GTSRB. The authors of [49] proposed an approach for TSDR using SegU-Net and a modified Tversky loss function With L1-Constraint that achieved 94.60% and 80.21% precision and recall, respectively, on the CURE-TSD dataset. Liu et al. [50] proposed traffic sign recognition and detection approaches, which first extract the region of interest and after verification of the traffic sign through an SVM classifier, it classifies the traffic sign into traffic sign categories. The proposed approach achieves the highest accuracy 94.81%.

Another study [51] used the Inceptionv3 model trained with transfer learning on the BelgiumTSC dataset, obtaining an accuracy of 99.18%. In [52], the authors found that the Tiny-YOLOv2 network is fast but outperformed by YOLOv2 or YOLOv3 deep learners. While the authors of [53] introduced real time image enhancement CNN and achieved an accuracy of 99.25% for the BelgiumTSC, 99.75% for GTSRB, and 99.55% for Croatian Traffic Sign (rMASTIF). Authors of [54], developed a real time TSR by using the You Only Look Once (YOLO) algorithm to train the model for Malaysian traffic sign recognition and tested it on five types of warning traffic signs. In [55] authors propose a lightweight CNN architecture for the recognition of the traffic sign GTSRB dataset, and they achieved 99.15% accuracy. In one another study [56], a novel semi supervised classification technique is adopted for TSR with weakly-supervised learning and self-training. An ensemble of CNN was used for the recognition of the traffic signs and achieved higher than 99% accuracy for the circular traffic signs of the German and BelgiumTSC datasets [57]. Lu et al. [58] use multi-modal tree structure embedded multitask learning for the GTSRB dataset and achieved an overall accuracy of 98.27%. In [59], the authors improved the VGG-16 deep model by removing some redundant convolutional layers and adding Batch Normalization and global average pooling layer to improve the performance of the network, while [60] proposed a hybrid 2D-3D CNN. In [61], the authors proposed a traffic sign recognition system that learns learning hierarchical features based on multi-scale CNNs. In one another study [62], the authors proposed a real-time TSDR for Chinese and German roads. In [63] authors proposed a robust custom feature extraction method and multilayer artificial neural network for the recognition of traffic signs in real time.

Additionally, a few works perform classification depending on multiple frames. In a study [64], authors considered the sequences of frames of the street view images and achieved an 87.03% evaluation score, i.e., the ratio of true positive and true positive + false positive + false negative. In another study, Yuan et al. [65] proposed a video based traffic sign detection and recognition mechanism to fuse the result of all frames for final classification. They utilized a multi-class SVM with two different fusion strategies, i.e., equal weighting and a scale based weighting scheme which achieved 99.48% accuracy on the TS2010 dataset.

In the literature, there are many studies [66,67,68,69] focusing on single frame TSR, and very few studies [64,65] that process multiple frames. According to our knowledge based on the literature review, there is no study available that considers the sliding windows approach for traffic sign recognition.

### 2.3. Background on Comparative Studies

Only a few comparative studies have been proposed in the literature. For example, Jo [15] trained different supervised classifiers on HOG features extracted from the GTSRB dataset. Similarly, Schuszter [70] reported on experiments with the BelgiumTSC dataset [32], where HOG features were extracted from images and then fed to the SVM to classify one of the six basic traffic sign subclasses. Yang et al. [19] provide a comparison of different classifiers, such as the K-NN, SVM, Random Forest and AdaBoost trained by using combinations of features. This study reported the highest accuracy by using Random Forest with the combination of LBP and HOG features. Another study [29] compared traditional supervised classifiers and deep learning models on three datasets, i.e., GTSRB, BelgiumTSC and DITS considering three broad categories of traffic signs, i.e., red circular, blue circular and red triangular. Noticeably, both traditional supervised classifiers and deep classifiers achieved perfect accuracy on GTSRB. Moreover, the authors of [18] trained different classifiers for traffic sign recognition. They considered the GTSRB dataset and extracted HOG features to train LDA and Random Forest. Additionally, they used the committee of Convolutional Neural Networks (CNN) and multiscale-scale CNN. While in the study [31] authors organized a competition to classify GTSRB dataset traffic signs. These traffic signs were categorized by human and ML algorithms and an accuracy of 98.98% was achieved which is comparable to human performance on this dataset.

## 3. Sliding Windows to Improve TSR

TSR naturally fits the analysis of sequences of images being collected as the vehicle approaches the traffic sign. Therefore, we organize a complex classifier that processes sliding windows of frames

**Figure 2:**
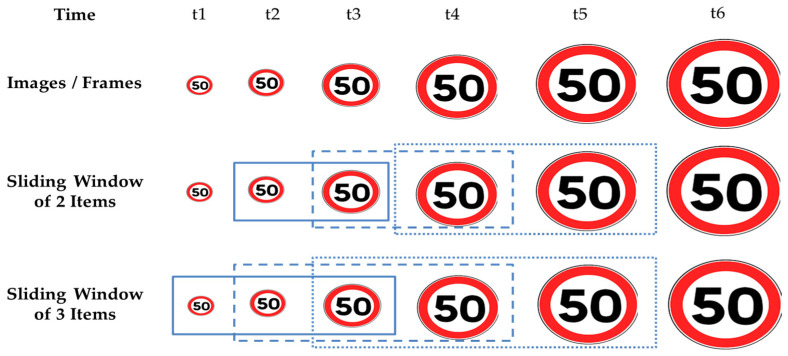
Example of Sliding Windows. Dotted, dashed and solid boxes show sliding windows, respectively, at t5, t4, t3.

As shown in Figure 2, a sliding window of size s contains (i) the most recent frame sampled by the sensors on the vehicle plus (ii) the s-1 most recent frames. The figure represents how sliding windows of size s = 2 and s = 3 evolve as time passes by as the vehicle approaches a speed limit sign. Intuitively, the closer the vehicle gets to the traffic sign, the more visible and clearer the traffic sign gets. On the other hand, the sooner the TSR correctly classifies a traffic sign, the better it is for the ADAS, e.g., it may provide more time for emergency braking, whenever needed.

### 3.1. Sliding Windows and Meta-Learning

Adopting sliding windows of s images calls for a rework of the TSR system. In particular, classification should be carried out using s subsequent classifications, which contribute to the final decision on the traffic sign. Those single-frame classifications for subsequent frames have to be combined by utilizing an independent strategy that delivers the result of this ensemble of single-frame classifiers.

Such a combination is usually orchestrated through meta-learning [30,71], which uses knowledge acquired during base-learning episodes, i.e., meta-knowledge, to improve classification capabilities. More specifically [72], a base-learning process starts feeding images into one or more base classifiers to create meta-data at the first stage. Results of those base learners, i.e., meta-data are provided alongside other features to the meta-level classifier as input features, which in turn provides the classification result of the whole meta-learner.

**Figure 3:**
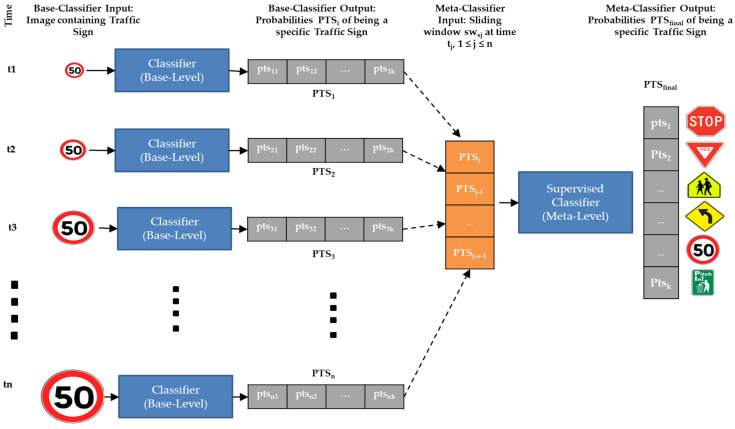
Diagram representing TSR which uses sliding windows. Blue solid boxes represent single-frame classifier in Figure 1.

The paradigm of meta-learning can be adapted to TSR as shown in Figure 3. Let k be the number of different categories of traffic signs (i.e., classes), and let s be the size of the sliding window. Starting from the left of the figure, frames are processed by means of single-frame base-level classifiers, which provide k probabilities PTS_i_ = {pts_i1_, … pts_ik_} to classify each frame. Depending on the current time t_j_, we create a sliding window of at most s×k items, namely swsj = {PTS_j_, PTS_j-1_, … PTS_j-s-1_}, which builds the meta-data to be provided to the meta-level classifier. On the right side of Figure 3, the meta-level classifier processes such meta-data and provides the k probabilities PTS_final_, which will constitute the classification result of the whole sequence within the sliding window. As time moves on, we will have newly captured images and the sliding window will process the most recent s × k items. Note, that the sliding window sw_sj_ may contain less than s × k items when j < s (e.g., the window of size 3 at time t2 in Figure 2). In those cases, the TSR system will decide based on a single-frame classification of the most recent image.

### 3.2. A Stacking Meta-Learner

The structure of the meta-learner we described previously is traditionally referred to as Stacking. Stacking [73] builds a base-level of different classifiers as base learners. Base-learners can be trained with the exact same training set or with different training sets, mimicking Bagging [74]. Each of the n base-learners generates meta-features (PTS_i_, 1 ≤ i ≤ n in Figure 3) that are fed to another independent classifier, the meta-level classifier, which calculates and delivers the final output (PTS_final_ in Figure 3).

In our instantiation of the Stacker, we use the same base-level classifier, which can either be a deep learner or a traditional supervised classifier but feed each base-learner with a different image. The meta-level classifier is necessarily a supervised (non-deep) classifier as it has to process numeric features contained in sw_sj_ rather than images.

### 3.3. Long Short-Term Memory Networks (LSTM)

As an alternative to stacking, we plan the usage of LSTM networks [75,76]. An LSTM network is a Recurrent Neural Network that learns the long-term dependencies between time steps of sequence data by orchestrating two layers. Those networks do not have a meta-learning structure as a stacker: however, they perfectly fit the analysis of sliding windows of traffic signs as they are intended to be used for the classification of sets or sequences by directly processing multiple frames. The first layer contains a sequence of inputs, which are then forwarded to the LSTM fully connected layer, and finally, the output layer shows the classification result.

## 4. Methodology, Inputs and Experimental Setup

This section describes the methodology, inputs, and experimental setup to compare single-frame classifiers and approaches built upon sliding windows, such as Stacking and LSTM networks. Results will be presented, analyzed, and discussed in Section 5.

### 4.1. Methodology for a Fair Comparison of TSR Systems

We orchestrate our experimental methodology as follows:

- **Datasets and Pre-processing.** Images go through a pre-processing phase to resize them to the same scale and enhance the contrast between background and foreground through histogram equalization.
- **Feature Extraction** (Section 4.3). Then, each pre-processed image is analyzed to extract features: these will be used with traditional supervised classifiers, while deep learners will be directly fed with pre-processed images.
- **Classification Metrics** (Section 4.4). Before exercising classifiers, we select metrics to measure the classification capabilities of ML algorithms which apply both to single-frame classifiers and to others based on sliding windows.
- **Single-Frame Classification.** Both supervised (Section 4.5) classifiers and deep learners (Section 4.6) will be trained and tested independently, executing grid searches to identify proper values for hyper-parameters.
- **Sliding Windows with Stacking Meta-Learners** (Section 4.7). Results of single-frame classifiers will then be used to build Stacking learners as described in Section 3.2 and by adopting different meta-level classifiers.
- **Sliding Windows with LSTM (**Section 4.8). Furthermore, sliding windows will be used to exercise LSTM networks as described in Section 3.3.

Exercising such methodology with its inputs required approximately 6 weeks of execution. The experiments were conducted on an Intel(R) Core (TM) i5-8350U CPU@1.7 GHz 1.9 GHz running MATLAB. MATLAB implementations of Deep Learners also use our NVIDIA Quadro RTX 5000 GPU.

### 4.2. TSR Datasets and Traffic Sign Categories

We conducted extensive research to identify commonly used labeled datasets reporting on sequences of traffic signs with overlapping categories. We selected three public datasets which report on sequences of images of traffic signs, namely: (i) the BelgiumTSC dataset [32], (ii) the GTSRB dataset [31], and (iii) the DITS [33]. Details about their structure and the categories of traffic signs are in Table 1 and Table 2, respectively.

#### 4.2.1. German Traffic Signs Recognition Benchmark Dataset

The German Traffic Signs Recognition Benchmark (GTSRB [31]) dataset is widely used in the literature [15,18,19,31] as it reports on images of traffic signs belonging to eight categories with heterogeneous illumination, occlusion and distance from the camera. The dataset contains sequences of 30 images for each traffic sign, which were gathered as the vehicle was approaching it. The authors made available 1307 training and 419 testing sequences of images for a total of 51,780 images contained in the dataset. Table 2 depicts examples of traffic signs for each category of traffic sign contained in this dataset. Importantly, the rectangular traffic signs we mapped into category 8 in the table do not appear in the GTSRB dataset but appear in other datasets considered in this study.

#### 4.2.2. BelgiumTSC Dataset

The BelgiumTSC dataset [32] is another dataset of traffic signs which was extensively used in the last decade [32,70]. The BelgiumTSC contains eight categories of traffic signs, shown from category 1 to category 8 in Table 2. The dataset is smaller than the GTSRB: the BelgiumTSC contains only 2362 sets of three images taken with different cameras from different viewpoints. It follows that this dataset reports triple images for each traffic sign which are all taken at the same time and thus are not time- ordered: this requires a dedicated discussion that we expand on in Section 5.4.

#### 4.2.3. Dataset of Italian Traffic Signs Dataset

The Dataset of Italian Traffic Signs (DITS) dataset is considered more challenging than others in the literature [33] as it contains traffic signs images that were taken under non-optimal lighting conditions, e.g., day, night-time, foggy weather. The DITS contains 623 sequences containing a varying, time-ordered, number of frames. We point out that DITS is the only dataset in this study that contains all the nine categories of traffic signs reported in Table 2 and as such, it provides a complete view of all potential traffic signs. The dataset contains 500 training sequences and 123 testing sequences of varying lengths as summarized in Table 1.

### 4.3. Feature Descriptors

In this study, we extract features from images by means of *handcrafted*, i.e., HOG, LBP and *deep*, i.e., AlexNet and ResNet, feature descriptors, as described below.

- **Histogram of Oriented Gradients (HOG)** mostly provides information about key points in images. The process partitions an image into small squares and computes the normalized HOG histogram for each key point in each square [17].
- **Local Binary Patterns (LBP)** encode local textures [16] by partitioning each image into non-overlapping cells. Then, LBP isolates local binary patterns and uses small gray-scale discrepancies to identify specific features. Its behavior is invariant to the monotonic transformation of grayscale.
- **AlexNet Features (AFeat)** are extracted through a pre-trained AlexNet [34], composed of five convolutional layers and three fully connected layers. Convolutional layers are basically extracting deep features from RGB images of size 227 × 227. We extract a feature vector of 4096 items by fetching data at the fully connected layer “fc7”.
- **ResNet Features (RFeat)** are extracted from a ResNet-18 [35], a convolutional neural network with 18 hidden layers. Convolutional layers are extracting deep features from RGB images of size 224 × 224 Similarly to AlexNet, we extract 512 features by extracting data at the global average pooling layer “pool5”.

In addition, we combine hand-crafted and deep feature descriptors that are consequently fed simultaneously to classifiers: couples as {HOG ∪ LBP}, {AFeat ∪ HOG}, {AFeat ∪ LBP}, {RFeat ∪ HOG}, {RFeat ∪ LBP}, {AFeat ∪ RFeat}, and triples of {AFeat ∪ HOG ∪ LBP} and {RFeat ∪ HOG ∪ LBP}.

### 4.4. Classification Metrics

The performance of classifiers for TSR is usually compared by means of classification metrics. These metrics are mostly designed for binary classification problems, but they can be adapted also to measure multi-class classification performance. Amongst the many alternatives, TSR mostly relies on accuracy [77,78], which measures the overall correct and incorrect classifications. Correct classifications reside in the diagonal of the confusion matrix, whereas any other item of the confusion matrix is counted as a misclassification.

It should be noticed that this is a quite conservative metric for TSR as it considers all misclassifications at the same level. Instead, we may not be too worried about misclassifying an informative sign (e.g., Category 8 in Table 2) with a stop sign, whereas the opposite represents a very dangerous event. That being said, for ease of comparison with existing studies, we calculate accuracy according to its traditional formulation, thus considering each misclassification as equally harmful.

### 4.5. Traditional Supervised Classifiers and Hyper-Parameters

Traditional Supervised classifiers process features extracted from images. Amongst the many alternatives, we summarize below those algorithms that frequently appear in most studies about TSR.

- **K Nearest Neighbors (K-NN)** algorithm [13] classifies a data point based on the class of its neighbors, or rather other data points that have a small Euclidean Distance with respect to the novel data point. The size k of the neighborhood has a major impact on classification, and therefore, needs careful tuning, which is mostly achieved through grid or random searches.
- **Support Vector Machines (SVMs)** [14], instead, separate the input space through hyperplanes, whose shape is defined by a kernel. This allows performing either linear or non-linear (e.g., radial basis function RBF kernel) classification. When SVM is used for multi-class classification, the problem is divided into multiple binary classification problems [79].
- **Decision Tree** provides a branching classification of data and is widely used to approximate discrete functions [36]. The split of internal nodes is usually driven by the discriminative power of features, measured either with Gini or Entropy Gain. Training of decision trees employs a given number of iterations and a final pruning step to limit overfitting.
- **Boosting (AdaBoostM2)** [39] ensembles combine multiple (weak) learners to build a strong learner by weighting the results of individual weak learners. Those are created iteratively by building specialized decision stumps that focus on “hard” areas of input space.
- **Linear Discriminant Analysis (LDA)** is used to find out the linear combination of features that efficiently separates different classes by distributing samples into the same type of category [38]. This process uses a derivation of Fisher discriminant to fit multi-class problems.
- **Random Forests** [37] build ensembles of Decision Trees, each of them trained with a subset of the training set extracted by random sampling with replacement of examples.

Each supervised algorithm has its own set of hyper-parameters. To such an extent, we identified the following parameter values to exercise grid searches.

- K-NN with different values of k, i.e., different odd values of k from 1 to 25. Additionally, we observe that DITS contains nine categories of traffic signs: therefore, we disregard the usage of k = 9 to further avoid ties.
- SVM: we used three different kernels: Linear, RBF and Polynomial (quadratic), leaving other parameters (e.g., nu) as default.
- Decision Tree: we used the default configuration of MATLAB which assigns MaxNumSplits = training sample size 1, with no depth limits on decision trees.
- Boosting: we created boosting ensembles with AdaBoostM2 by building 25, 50, 75, and 100 trees (decision stumps) independently.
- Random Forest: we build forests of 25, 50, 75, or 100 decision trees.
- LDA: we Trained LDA using different discriminants, namely: pseudo-linear, diag-linear, diag-quadratic, and pseudo-quadratic.

### 4.6. Deep Learners and Hyper-Parameters

Deep learners may be either built from scratch or more likely—by adapting existing models to a given problem through transfer learning (i.e., knowledge transfer). Through transfer learning, we fine tune the fully connected layers of the deep model, letting all convolutional layers remain unchanged. Commonly used deep learners for the classification of images and object recognition are below.

- **AlexNet** [34] is composed of eight layers, i.e., five convolutional layers and three fully connected layers that were previously trained on the ImageNet database [80], which contains images of 227 × 227 pixels with RGB channels. The output of the last fully connected layer is provided to the SoftMax function, which provides the distribution of overall categories of images.
- **InceptionV3** is a deep convolutional neural network built by 48 layers that were trained using the ImageNet database [80], which includes images (299 × 299 with RGB channels) belonging to 1000 categories. InceptionV3 builds on (i) the basic convolutional block, (ii) the Inception module and finally (iii) the classifier. A 1x1 convolutional kernel is used in the Inceptionv3 model to accelerate the training process by decreasing the number of feature channels; further speedup is achieved by partitioning large convolutions into small convolutions [40].
- **MobileNet-v2** [41] embeds 53 layers trained on ImageNet database [80]. Differently from others, it can be considered a lightweight and efficient deep convolutional neural network with fewer parameters to tune for mobile and embedded computer vision applications. MobileNet-v2 embeds two types of blocks: the residual block and a downsizing block, with three layers each.

Those deep learners can be tailored to TSR through transfer learning. Fully connected layers are trained on defined categories of traffic signs with different learning rates (LR) to fine-tune the models which are already trained on the ImageNet database of 1000 categories. Additionally, we employ data augmentation to avoid model overfitting; this was conducted through X and Y translation with a random value between [−30, 30] and scale range within a range [0.7, 1].

The hyper-parameter learning rate controls how fast weights are updated in response to the estimated errors, and therefore, controls both the time and the resources needed to train a neural network. Choosing the optimal learning rate is usually a tricky and time-consuming task: learning rates that are too big may result in fast but unstable training, while small learning rates usually trigger a heavier training phase which may even get stuck without completing correctly. In our experiments, we varied learning rate as follows: {0.05, 0.01, 0.005, 0.001, 0.0005, 0.0001} for Inceptionv3 and MobileNet-v2, and {0.0001, 0.0005, 0.00001, 0.00005, 0.000005, 0.000001} for AlexNet, which resulted in very low accuracy when using the same learning rates of the Inceptionv3 and MobileNet-v2. Noticeably, training a deep classifier with the highest learning rate in the interval reduce the training time with respect to using the smallest value in the interval (e.g., training Inceptionv3 with a learning rate of 0.05 instead of using 0.0001).

We set a minimum batch size of 32, with 10 train epochs and stochastic gradient descent with momentum (sgdm) optimizer for all the experiments on each dataset to fine-tune the models for TSR. Furthermore, we used the loss function ‘crossentropyex’ at the classification layer and the fully connected weights and biases were updated with a learning factor (different from learning rate) of 10. We had the weights vector size associated with the last fully connected layers [Num_cat × 4096], [Num_cat × 1280], and [Num_cat × 2048] for Alexnet, MobileNet-v2 and Inceptionv3 models, respectively, where Num_cat represented the number of traffic sign categories in each dataset.

### 4.7. Stacking Meta-Level Learners

Stacking meta-learners orchestrate a set of base-learners, which provide meta-data to the meta-level learner. In our study, we foresee the usage of different meta-level learners as listed below.

- **Majority Voting** [42] commits the final decision based on the class the majority of base-learners agree upon. This technique is not very sophisticated, albeit it was and is widely used to manage redundancy in complex systems [81] and to build robust machine learners [82].
- **Discrete Hidden Markov Model (DHMM)** [43]. For each class, a separate Discrete HMM returns the probability of an image belonging to that class. The classification result of the frames within the sliding window is given as input to all three DHMMs. Each DHMM returns the likelihood of the sequence to a specific class. The higher the likelihood to a specific class is decided as a final label for that specific sequence.
- **Supervised Classifiers in Section 4.5.** These classifiers can be employed as meta-level learners as meta-data resembles a set of features coming from base-learning episodes.

The parameters we used to execute grid searches and train meta-level learners above are as follows.

- Majority Voting: no parameter is needed.
- Each DHMM model was trained with 500 iterations.
- Supervised Classifiers: we used the same parameter values we already presented in Section 4.5.

### 4.8. Long-Short Term Memory (LSTM) Networks

LSTM networks are artificial recurrent neural networks, which efficiently process sequences of images, and therefore, suit the classification of sequences of traffic signs. LSTM networks are trained on all 12 feature sets in Section 4.3 independently considering three different training functions or optimizers, i.e., ‘adam’, ‘sgdm’, and ‘rmsprop’ with a learning rate of 0.001.

## 5. Results and Discussion

This section reports and discusses the results of our experimental campaign. We split the results into two sub-sections: Section 5.1 describes the experimental results of single-frame classifiers, while Section 5.2 reports on the results achieved by classifiers that consider sliding windows of frames.

### 5.1. TSR Based on Single Frame

First, we elaborate on the classification performance of TSR systems that process frames individually.

#### 5.1.1. Highest Accuracy for Each Dataset

**Figure 4:**
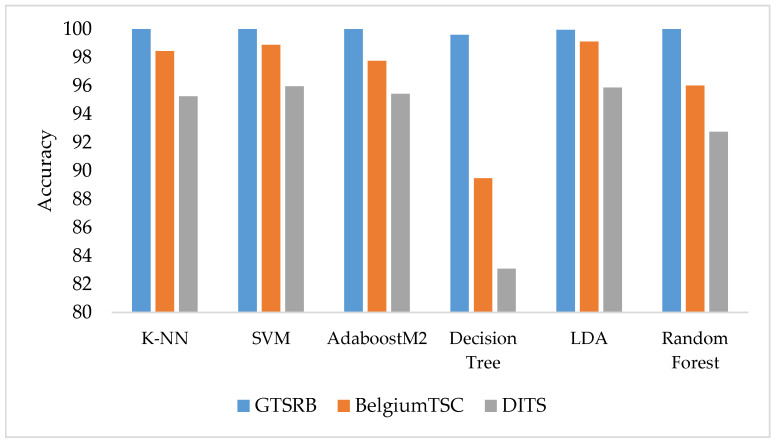
Highest accuracy achieved by traditional supervised classifiers on each dataset.

Figure 4 depicts a bar chart diagram reporting the highest accuracy achieved by classifiers in each of the three datasets. It is clear from the blue solid bars in Figure 4 that almost all classifiers give better performance on the GTSRB dataset compared to the other two datasets, i.e., BelgiumTSC and DITS. All classifiers in the figure but Decision Tree and LDA achieve perfect accuracy on the GTSRB dataset. The reason behind the high accuracy may be the higher number of training samples and better image quality of the GTSRB dataset compared to the other two datasets. Instead, SVM provides the highest accuracy of 95.94% in DITS, with LDA that comes close at 95.85%. Consequently, the highest accuracy in each dataset is not always achieved by the same algorithm, despite K-NN, SVM and LDA performing better overall compared to other supervised classifiers.

#### 5.1.2. Impact of Feature Descriptors

Table 3 further elaborates on the impact of features on accuracy scores achieved by supervised classifiers on each dataset. Supervised classifiers achieve perfect accuracy with all feature descriptors on GTSRB. Instead, the combination of AFeat and RFeat builds a feature descriptor that allows algorithms to achieve the highest accuracy of 95.94% for DITS and 99.12% for BelgiumTSC. Additionally, AFeat and RFeat descriptors provide features that allow algorithms to reach higher accuracy. By using just a single feature descriptor AFeat always achieves the highest accuracy on all three datasets, while the second highest accuracy is achieved by RFeat. Instead, using only LBP, HOG or their combination generates accuracy scores that are lower than potential alternatives. Moreover, it is worth noticing how combining feature descriptors provides features that increase the classification performance of supervised classifiers, such as: from 95.51% to 95.94% in DITS, and from 98.84% to 99.12% in BelgiumTSC.

#### 5.1.3. Results of Deep Classifiers

We explore the results of the deep classifiers considered in this study with the aid of Table 4, which shows accuracy scores achieved by those classifiers for different learning rates.

MobileNet-v2 achieves the highest accuracy out of the three deep learners for the GTSRB dataset with a learning rate of 0.001, whereas a learning rate of 0.00005 maximizes the accuracy scores of AlexNet on the BelgiumTSC dataset. Instead, the learning rate of 0.0001 allows InceptionV3 to reach the maximum accuracy of 96.03% for the DITS dataset, outperforming MobileNet-v2 and AlexNet, which instead achieves the highest accuracy in the BelgiumTSC dataset with a learning rate of 0.0005. Interestingly, whereas accuracy scores for GTSRB do not vary a lot when using different learning rates, the choice of the learning rate becomes of paramount importance when classifying DITS and BelgiumTSC datasets. Particularly, the bottom of Table 4, the third column, shows a 14.97% accuracy on the BelgiumTSC dataset using learning rates of 0.05 and 0.005, which is a very poor achievement. For these learning rates, the training process was unstable, with weights that were updated too fast and ended up with a classifier that has semi-random classification performance. Unfortunately, we could not identify a single deep classifier that outperforms others in all three datasets.

### 5.2. TSR Based on Sliding Windows

This section elaborates on the classification performance of TSR systems that process a sliding window of multiple frames.

#### 5.2.1. Meta Learning with Traditional Base Classifiers

Table 5 reports scores achieved by stacking meta-learners built using (i) the three traditional supervised classifiers that performed better in Section 5.1.1 as base learners, and (ii) different meta-level learners, such as K-NN, SVM, LDA, Decision Tree, Majority Voting, Boosting, Random Forest and DHMM. The GTSRB dataset does not appear in Table 5 since single-frame traditional classifiers alone already achieved perfect classification. The table reports the highest accuracy scores achieved by each stacking meta-level classifier by using different combinations of base-learners (K-NN, SVM, LDA) and window sizes of two and three items.

Overall, LDA as a base-level classifier with a K-NN meta-level classifier is the preferred choice (bolded values in Table 5) on DITS and on BelgiumTSC with a sliding window of three items. Instead, using ensembles of Decision Trees as AdaBoost and Random Forests sparingly gives very low accuracy scores (see italicized numbers in the 10th and 11th columns of Table 5), showing that those two classifiers do not always adequately play the role of a meta-level classifier for a stacker.

Results for DITS in Table 5 show that using a sliding window of three items generally improves accuracy with respect to using a sliding window of only two items. A sliding window of three items allowed stacking meta-learners, which used K-NN or LDA as meta-level classifiers to reach perfect accuracy (100%) on the DITS dataset using either LDA or SVM as base-learners. This result was largely expected: the more information is available (i.e., wider sliding window), the fewer misclassifications we expect from a given classifier.

Instead, we obtained maximum accuracy for the BelgiumTSC by using a sliding window of two items, whereas using three items often degrades classification performance. At a first glance, this result is counter-intuitive with respect to previous discussions. However, the reader should note that the BelgiumTSC dataset reports on a set of images of the same traffic signs which are captured with multiple input cameras without any temporal order. Consequently, the sliding window for the BelgiumTSC contains images of the traffic sign which are taken from different angles and may lead the meta-learner to lean towards misclassifications rather than improving accuracy. In fact, for this dataset, there is no direct relation between the size of the window and accuracy values, which instead turned out to be evident for the other datasets.

#### 5.2.2. Meta Learning with Base-Level Deep Classifiers

Table 6 has a structure similar to Table 5 but employs base-level deep classifiers to build the stacking meta-learner, and also reports on all datasets as deep classifiers based on a single frame but did not achieve perfect accuracy on any of the three datasets. Deep base-level classifiers in conjunction with K-NN as a meta-level classifier achieved perfect classification on all three datasets, as shown by bold values in Table 6. GTSRB turns out to be the dataset that provides the higher average accuracy by using a different base and meta-level classifiers. The highest achieved accuracies are highlighted in Table 6 with bold typeset. It is very interesting to discuss that all three deep learning models (base-level classifiers) with meta-level classifiers K-NN, LDA, Boosting and Random Forest give 100% accuracy, while MobileNet-v2 achieves 100% accuracy with all meta-level classifiers for a sliding window of size 2 or 3 on the GTSRB dataset. Inceptionv3 and MobileNetv2 with meta-level classifiers (K-NN, AdaboostM2) achieve 100% accuracy on the DITS dataset for sliding windows of size 2 and 3, respectively, While AlexNet base-level classifier with Majority voting and K-NN as the meta-level classifier achieves 100% accuracy for both sliding windows of size 2 & 3 on BelgiumTSC dataset.

Similarly, to Table 5, we observe that AdaboostM2 does not show up as a reliable meta-level classifier as it provides very low accuracy for the BelgiumTSC with a sliding window of three frames. All meta-level classifiers with base-level classifier Mobilenet-v2 achieve 100% accuracy on the GTSRB dataset, whose sequences contain 30 images of the same traffic sign, and therefore, provide much information for the stacking classifier to classify traffic signs as the window slides.

#### 5.2.3. Results of LSTM Networks

Table 7 reports accuracy scores of LSTM networks on the BelgiumTSC and DITS datasets with a sliding window of size 2 or 3. Similarly to Section 5.2.1, we omit the GTSRB dataset since it is perfectly classified by single-frame traditional classifiers. We independently trained the LSTM by using each of the 12 feature sets in Section 4.3, with different window sizes (WS) and by using three different optimizers: adam, sgdm and rmsprop.

Table 7 reports the highest accuracy achieved by LSTM by using a given WS and optimizer function. It is evident how the adam optimizer always allows achieving the highest accuracy scores in both datasets and with different WS. Additionally, accuracy is always higher when using a window of size 3 with respect to a window containing only two items: this was expected for DITS, whose images are time-ordered, but it is also verified for the BelgiumTSC, which does not have such ordering. Overall, the results of the LSTM are slightly lower than stacking meta-learners using traditional base-level classifiers and clearly worse than stacking using deep base-level classifiers, which achieves perfect accuracy on all datasets.

### 5.3. Comparing Sliding Windows and Single-Frame Classifiers

Independent analyses and discussions of results in Section 5.1 and Section 5.2 provided interesting findings concerning both traditional supervised and deep base-level classifiers and the usage of sliding windows to improve the classification performance through meta-learning.

Traditional supervised classifiers, such as K-NN, SVM, AdaboostM2, and Random Forests achieved a perfect classification of each image contained in the GTRSB dataset. Moreover, we observed how combining deep features descriptor {AFeat ∪ RFeat} allowed traditional classifiers to reach the highest accuracy in any of the three datasets, achieving 100%, 95.94%, 99.12% on the GTSRB, DITS and BelgiumTSC datasets, respectively. On the other hand, deep classifiers outperform traditional classifiers on the DITS and BelgiumTSC datasets but still cannot reach a perfect classification accuracy.

Noticeably, stacking meta-learners that take advantage of sliding windows achieve perfect classification accuracy on all three datasets when using deep base-level classifiers and K-NN as meta-level classifiers. These results show that orchestrating sliding windows critically increases the classification performance compared to single frame classifiers. Differently, LSTM networks achieve 97.56% and 99% of accuracy on the DITS dataset for a sliding window of size 2 or 3, respectively, which is better than single frame classifier performance, but still inferior with respect to stacking meta-learners.

**Figure 5:**
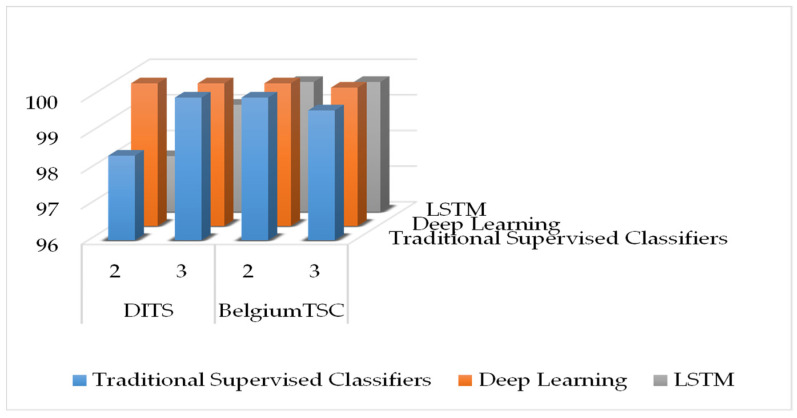
Highest accuracy achieved by LSTM, stacker with supervised base-level, and stacker with deep base-level on BelgiumTSC and DITS.

Figure 5 compares the accuracy achieved by stacking meta-learners and LSTM networks by means of a bar chart. Base-level traditional supervised classifiers with stacking meta learners achieved 98.37% and 100% accuracy on the DITS dataset considering a sliding window of two and three inputs, respectively, which is slightly higher than the LSTM scores. A similar trend can be observed for the BelgiumTSC, while the GTSRB scores are not reported in the chart as it does not require sliding windows to achieve perfect accuracy.

### 5.4. In-Depth View of BelgiumTSC

Similarly, to the GTSRB and DITS, we observed perfect classification by using a stacker with deep base-level classifiers also with the BelgiumTSC dataset, which contains unordered sets of images rather than sequences. Consequently, our meta-learning strategy proves to be beneficial even if images in the sliding window are not time-ordered.

**Figure 6:**
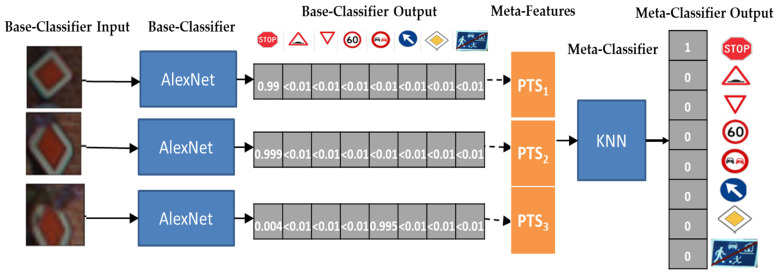
Instantiation of the stacking-meta learner with AlexNet base-learner and SVM meta-level learner, managing a sliding window of size 3 for BelgiumTSC. The three frames we use as input describe a Diamond sign (Category 7) which is misclassified using all three frames.

However, Table 7 showed that a sliding window of three items performs poorly with respect to using only two items, which may seem counterintuitive. Figure 6 shows one of those cases in which using a window of two items is beneficial with respect to using three items. The upper part of Figure 6 represents the process adopted for the classification of a Diamond traffic sign (Category 7) when using a window of three images. All the three images taken from different viewpoints are individually classified by the base-level classifier AlexNet, which returns the probabilities PTS of belonging to all classes (see Base-Classifier output in the figure). These three probability vectors (which match the PTS_i_ in Section 3.2) are fed to the meta-level classifier to commit the final decision. We observe that PTS_1_ and PTS_2_ give almost a certain probability of belonging to class 7 (0.999), while PTS_3_ gives a higher probability for class 1 (i.e., stop traffic sign). With those results, the SVM meta-learner decides that the traffic sign is a stop sign, ending up with a misclassification. Clearly, the third image is taken from a different angle, has some blurring and makes the meta-learner lean towards a misclassification rather than helping.

**Figure 7:**
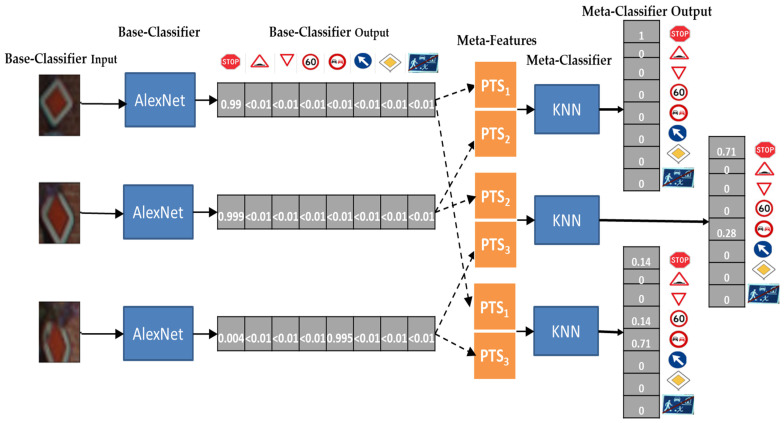
Instantiation of the Stacking-Meta learner with AlexNet Base-learner and SVM meta-level learner, managing a sliding window of size 2 for the BelgiumTSC. The three frames we use as input describe a Diamond sign (Category 7) which is misclassified using all three frames (Figure 6) but may be classified correctly by using a shorter window.

Instead, Figure 7 shows the process to classify the same inputs using a window of two items. When {PTS_1_, PTS_2_} are provided as meta features to a meta-level classifier, the final output shows a high likelihood of being a category 7 which is indeed a correct classification. Meanwhile, providing {PTS_2_, PTS_3_} or {PTS_1_, PTS_3_} as meta features lead the stacker to misclassify the set of images as a stop sign (category 1): the predicted final output is class 6 which is a wrong prediction. This enforces the conjecture that in this case using the third image constitutes noise that causes misclassification.

### 5.5. Timing Analysis

This section expands on the time required for classification using the different setups in this paper. Table 8 reports the average and standard deviation of time required for (i) feature extraction, (ii) single-frame classification, and (iii) stacking meta-learning across test images of three datasets.

Starting from feature extraction on the left of the table, it turns out that the extraction of handcrafted features takes slightly less time compared to deep features. However, even extracting deep features through ResNet-18 from a single image does not require on average more than 0.04 s (roughly 40 ms). Instead, the time required for exercising single-frame TSR classifiers varies a lot: traditional supervised classifiers need at most 200 ms to classify a given input set, whereas deep classifiers need more than half a second to classify an image with our hardware setup, depending on the number of layers of deep models. Indeed, the reader should note that whereas deep classifiers embed feature extraction through convolutional layers, traditional classifiers have the prerequisite of feature extraction. In fact, on the right of Table 8, we show that a TSR system that relies on AFeat ∪ RFeat features (i.e., most useful ones according to Table 3) provided to an SVM classifier takes on average 0.1974 s to classify an image: this includes feature extraction and classification itself. A perfect parallelization of the feature extractors cuts down this timing to 0.1756 and will be easily achievable on basic multi-core systems.

Table 8 also shows the time needed to perform other TSR strategies we discussed in this paper. Particularly, the third to sixth line on the right of the table show the time needed to classify an image using a sliding window of two or three items with different base-levels and meta-level learners. The time required for base-level learning equals single-frame classification: only the most recent frame in the window is processed, whereas probabilities assigned by classifiers to older frames are stored, and therefore, do not need to be re-computed again. The table reports on different base-learners but always uses K-NN as the meta-level learner, as this was the classifier that allowed reaching high scores in Section 5.2. K-NN takes on average 0.188 to classify a sliding window of two items, (i.e., two PTS vectors of 8/9 numbers each), and only slightly more time to process a sliding window of three items.

Overall, we can observe how most TSR systems that embed sliding windows are able to classify a new image in less than a second, whereas heavier deep learners make classification time lean towards two seconds. We believe that such timing performance albeit slower than using single-frame classifiers is still efficient enough to be installed on a vehicle, which only rarely samples more than a frame per second for TSR tasks. Nevertheless, using more efficient hardware, especially GPUs, could help in reducing, even more, the time required for classification.

### 5.6. Comparison to the State of the Art TSR

Ultimately, we recap the accuracy scores achieved by studies we already referred to as related works in Section 2.2 and Section 2.3, to compare their scores with ours. Therefore, Table 9 summarizes those studies, the datasets they used, and the accuracy they achieved. At a first glance, those studies conclude that their single-frame classifiers are often far from perfect classification. In fact, even in this study, we observed that single-frame TSR in the BelgiumTSC and DITS datasets cannot reach perfect accuracy (i.e., second-last row in the table). Unfortunately, promising studies [64,65], which describe multi-frame classifiers, do not rely on our datasets, and therefore, we cannot directly compare them.

To summarize, our experiment ended up achieving perfect classification on all datasets thanks to sliding windows (see last row of Table 9), dramatically improving existing studies on those datasets, for which perfect accuracy was hardly achieved by existing studies.

## 6. Concluding Remarks

To conclude the paper, we report in this section the limitations to the validity of our study, we summarize the findings and lessons learned in this paper and ultimately discuss future works.

### 6.1. Limitations to Validity

We report here possible limitations to the validity and the applicability of our study. These are not to be intended as showstoppers when considering the conclusions of this paper. Instead, they should be interpreted as boundaries or possible future implications which may impact the validity of this study.

#### 6.1.1. Usage of Public Data

The usage of public image datasets and public tools to run algorithms was a prerequisite of our analysis, to allow reproducibility and to rely on proven-in-use data. However, the heterogeneity of data sources and their potential lack of documentation may limit the understandability of data. In addition, such datasets are not under our control; therefore, possible actions, such as changing the way data is generated, are out of consideration. For example, creating longer sequences of traffic signs or creating a time-sequenced version of the BelgiumTSC is not possible at all.

#### 6.1.2. Parameters of Classifiers

Each classifier relies on its own parameters. Finding the optimal values of parameters is a substantial process that requires sensitive analyses and is directly linked with the scenario in which the classifier is going to be exercised. When applying classifiers to different datasets it is not always possible to precisely tune these parameters: instead, in this study, we perform grid searches, which run a classifier with different parameter values and choose the parameter that maximizes accuracy. This does not guarantee finding the absolute optimum value of a parameter for a given classifier on a given dataset but constitutes a good approximation [84].

### 6.2. Lessons Learned

This section highlights the main findings and lessons learned from this study.

- We observed that classifying images in the DITS dataset is harder than classifying the BelgiumTSC and GTSRB datasets as both base-level traditional supervised and deep classifiers’ performances are low comparatively. This is mostly due to the amount of training images and their quality, which is higher in the GTSRB compared to the other two datasets.
- Combining feature descriptors allows for improving classification performance. Particularly, we found that the {AFeat ∪ RFeat} descriptor allows traditional classifiers to maximize accuracy.
- Single-frame traditional supervised classifiers achieved perfect classification on the GTSRB dataset, while on the BelgiumTSC and DITS they show a non-zero amount of misclassifications. To the best of our knowledge, this result is due to the number of training samples, which is higher in the GTSRB with respect to the BelgiumTSC and DITS, and image quality, which again is better for the GTSRB. On the other hand, we achieved 100% accuracy by adopting a sliding windows based TSR strategy on all three considered datasets.
- There is no clear benefit in adopting deep classifiers over traditional classifiers for single-frame classification as they show similar accuracy scores. Additionally, both are outperformed, when using sliding windows for TSR.
- LSTM networks often, but not always, outperform single-frame classifiers but show lower accuracy than stacking meta-learners in orchestrating sliding windows.
- A stacking meta-learner with deep base-level classifiers and K-NN as meta-level classifier can perfectly classify traffic signs on all three datasets with any window size WS ≥ 2.
- For datasets that contain sequences (time-series) of images, enlarging the sliding window never decreases accuracy and, in most cases, raises the number of correct classifications.
- Deep learning models require more time compared to traditional supervised classifiers, especially because there are many layers, e.g., InceptionV3.
- Sliding windows based classification takes more time compared to single-frame classifiers but has remarkably higher classification performance across all three datasets.
- Overall, adopting classifiers that use a sliding window rather than a single-frame classifier allows reducing misclassifications, consequently raising accuracy.

### 6.3. Current and Future Works

Our study showed how the adoption of a stacking meta-learner in conjunction with sliding windows allows achieving perfect classification on the public GTSRB, BelgiumTSC and DITS datasets. Those datasets contain images taken in different parts of the world and mostly taken in semi-ideal lighting and environmental conditions. Therefore, they may not completely represent what a real TSR system installed on a vehicle will face during its life. As a result, we plan to explore the robustness of classifiers used in this study by injecting different types of faults/perturbations in the captured images [85], tracking the likely growth of misclassifications of individual classifiers. After this test, we plan to re-train (either from scratch or through transfer learning) classifiers using both original images from datasets and those faulty images. Furthermore, we plan to inject adversarial attacks to traffic sign images and using them both (i) as a test set, to observe the degradation of accuracy (if any) when processing corrupted frames, and (ii) during training, to learn a more reliable model. We believe that this process will allow us to build robust classifiers with very high accuracy, even when classifying faulty, adversarial, or corrupted images.

## Figures and Tables

**Table 1:** Details of the three datasets used in this study.

Dataset	Train Images	Test Images	Images per Sequence	Training Sequences	Test Sequences
**GTSRB**	39210	12570	30	1307	419
**DITS**	7500	1159	15	500	123
**BelgiumTSC**	4581	2505	3	1527	835

**Table 2:** Categorization of Traffic Signs into 9 categories based on their shape, color, and content.

Category	1	2	3	4	5	6	7	8	9
Traffic Signs	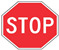	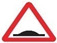	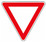	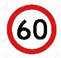	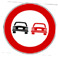	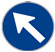	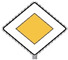	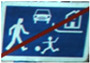	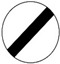
GTSRB	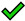	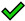	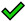	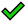	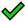	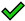	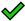	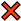	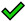
BelgiumTSC								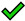	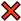
DITS								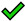	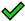

**Table 3:** Highest accuracy achieved using different feature descriptors on each dataset (bold highlighted values represent the highest achieved accuracy across each dataset).

Feature Descriptor (s)	GTSRB	DITS	BelgiumTSC
AFeat	**100.00**	95.51	98.84
RFeat	**100.00**	94.13	97.76
LBP	**100.00**	79.98	93.49
HOG	**100.00**	87.92	96.24
HOG ∪ LBP	**100.00**	88.26	96.56
AFeat ∪ RFeat	**100.00**	**95.94**	**99.12**
AFeat ∪ HOG	**100.00**	95.68	98.96
AFeat ∪ LBP	**100.00**	95.85	98.96
RFeat ∪ HOG	**100.00**	95.51	98.72
RFeat ∪ LBP	**100.00**	95.85	98.80
AFeat ∪ HOG ∪ LBP	**100.00**	95.42	98.88
RFeat ∪ HOG ∪ LBP	**100.00**	95.34	98.84

**Table 4:** Accuracy achieved by deep learners for each of the three datasets with varying learning rates (bold highlighted values represent the highest achieved accuracy across each dataset by deep classifiers).

	InceptionV3		MobileNet-v2		AlexNet	
	LR	Acc	LR	Acc	LR	Acc
**GTSRB**	0.01	96.62	0.01	96.11	0.0001	95.98
	0.05	93.56	0.05	93.38	0.0005	94.92
	0.001	96.95	0.001	**99.35**	0.00001	94.86
	0.005	97.06	0.005	96.42	0.00005	95.64
	0.0001	96.81	0.0001	96.83	0.000001	95.83
	0.0005	**98.03**	0.0005	96.65	0.000005	**96.07**
**DITS**	0.01	80.06	0.01	93.52	0.0001	87.40
	0.05	80.67	0.05	85.93	0.0005	86.45
	0.001	88.17	0.001	94.99	0.00001	**95.51**
	0.005	84.98	0.005	88.78	0.00005	92.06
	0.0001	**96.03**	0.0001	95.77	0.000001	92.23
	0.0005	91.88	0.0005	**95.94**	0.000005	95.16
**BelgiumTSC**	0.01	89.58	0.01	97.16	0.0001	99.24
	0.05	14.97	0.05	94.49	0.0005	92.57
	0.001	98.12	0.001	98.72	0.00001	99.52
	0.005	14.97	0.005	94.73	0.00005	**99.72**
	0.0001	99.64	0.0001	**99.24**	0.000001	97.92
	0.0005	**99.68**	0.0005	98.96	0.000005	99.24

**Table 5:** Results achieved using different meta-learners considering traditional classifiers as base classifiers varying window size (WS). We bolded the highest achieved accuracy using different combinations across each dataset and low accuracy achieved through AdaBoostM2 and Random Forest are italicized in the 10th and 11th columns.

Dataset	Base-Level Classifier	Single Frame Accuracy	WS	Stacking Meta-Level Classifier							
				Majority Voting	K-NN	SVM	LDA	Decision Tree	AdaBoostM2	Random Forest	DHMM
**BelgiumTSC**	KNN	98.44	2	99.40	99.04	98.8	98.80	98.68	98.80	*51.86*	98.56
	SVM	98.88		99.52	99.64	**99.76**	98.68	99.64	99.28	98.80	99.04
	LDA	99.12		99.64	99.40	99.28	99.28	98.32	98.92	97.84	99.40
	KNN	98.44	3	99.40	99.40	99.04	98.92	98.44	98.32	63.47	98.20
	SVM	98.88		99.52	99.52	**99.64**	99.40	98.56	*14.97*	99.28	98.80
	LDA	99.12		**99.64**	**99.64**	99.40	98.2	99.28	97.96	98.32	98.80
**DITS**	KNN	95.25	2	97.56	97.56	97.56	96.75	97.56	97.56	82.93	96.75
	SVM	95.94		96.75	97.56	**98.37**	97.56	95.12	*31.71*	95.12	95.93
	LDA	95.85		**98.37**	**98.37**	97.56	97.56	**98.37**	95.93	96.75	96.75
	KNN	95.25	3	99.00	99.00	99.00	99.00	99.00	99.00	85.00	98.00
	SVM	95.94		99.00	**100.00**	99.00	99.00	97.00	*36.00*	97.00	96.00
	LDA	95.85		99.00	**100.00**	98.00	**100.00**	99.00	98.00	99.00	98.00

**Table 6:** Results achieved using different meta learners with deep learners as base classifiers with varying window size (WS). We bolded the perfect classification (100% accuracy).

Dataset	Base-Level Classifier	Single Frame Accuracy	WS	Majority Voting	K-NN	SVM	LDA	Decision Tree	AdaBoostM2	Random Forest	DHMM
**BelgiumTSC**	AlexNet	99.72	2	99.88	**100.00**	99.64	99.88	99.88	99.40	99.16	99.76
	InceptionV3	99.68		99.88	99.88	99.64	99.88	99.52	99.88	99.64	99.64
	MobileNetv2	99.24		99.52	99.88	99.64	99.04	99.64	99.52	98.68	99.64
	AlexNet	99.72	3	**100.00**	**100.00**	99.28	99.88	99.88	99.76	99.76	99.28
	InceptionV3	99.68		99.88	99.88	99.40	99.52	99.40	*14.97*	99.76	99.52
	MobileNetv2	99.24		99.88	99.88	98.80	99.16	99.40	*14.97*	99.76	99.52
**DITS**	AlexNet	95.51	2	96.75	97.56	97.56	97.56	96.74	96.74	96.74	98.37
	InceptionV3	96.03		98.37	**100.00**	97.56	98.37	98.37	96.74	95.93	99.19
	MobileNetv2	95.94		97.56	99.18	99.18	99.18	99.18	**100.00**	98.37	99.19
	AlexNet	95.51	3	97.00	99.00	99.00	99.00	99.00	99.00	99.00	99.00
	InceptionV3	96.03		98.00	**100.00**	99.00	98.00	99.00	**100.00**	99.00	98.00
	MobileNetv2	95.94		98.00	**100.00**	99.00	**100.00**	99.00	**100.00**	**100.00**	**100.00**
**GTSRB**	AlexNet	96.07	2	97.37	**100.00**	99.76	**100.00**	98.09	**100.00**	**100.00**	98.09
	InceptionV3	98.03		**100.00**	**100.00**	**100.00**	**100.00**	**100.00**	**100.00**	**100.00**	99.76
	MobileNetv2	99.35		**100.00**	**100.00**	**100.00**	**100.00**	**100.00**	**100.00**	**100.00**	**100.00**
	AlexNet	96.07	3	0.9737	**100.00**	99.76	**100.00**	98.09	**100.00**	**100.00**	98.09
	InceptionV3	98.03		**100.00**	**100.00**	**100.00**	**100.00**	**100.00**	**100.00**	**100.00**	99.76
	MobileNetv2	99.35		**100.00**	**100.00**	**100.00**	**100.00**	**100.00**	**100.00**	**100.00**	**100.00**

**Table 7:** Accuracy of LSTM with window sizes 2 and 3.

Dataset	WS	Optimizer		
		adam	sgdm	rmsprop
**DITS**	**2**	**97.56**	**97.56**	96.74
**DITS**	**3**	**99.00**	**99.00**	98.00
**BelgiumTSC**	**2**	**99.40**	99.16	99.16
**BelgiumTSC**	**3**	**99.64**	99.28	99.40

**Table 8:** Time required for (left) feature extraction, (middle) exercising individual classifiers, and (right) different TSR strategies, either sequential or parallel execution.

Feature Extractor	Time in Seconds Avg ± St. Dev	Individual Classifier	Time in Seconds Avg ± St. Dev	TSR Strategy	Average Time in Seconds	
					(Sequential)	(Parallel)
HOG	0.0204 ± 0.0024	SVM	0.1344 ± 0.0364	Single-frame (AFeat ∪ RFeat + SVM)	0.1974	0.1756
LBP	0.0196 ± 0.0023	KNN	0.1205 ± 0.0302	Single-frame (InceptionV3)	1.4205	1.4205
AFeat	0.0218 ± 0.0023	LDA	0.1034 ± 0.0256	Stacking with WS = 2 (AFeat ∪ RFeat + SVM + KNN)	0.4036	0.3636
RFeat	0.0412 ±0.0034	InceptionV3	1.4205 ± 0.6613			
		MobileNetV2	0.6391 ± 0.2180	Stacking with WS = 2 (AlexNet + KNN)	0.5621	0.5621
		AlexNet	0.3749 ± 0.1407	Stacking with WS = 2 (InceptionV3 + KNN)	1.6085	1.6085

**Table 9:** Comparison with state of the art approaches.

Studies	Sequences of Frames	Achieved Accuracy (%)		
		GTSRB	BelgiumTSC	DITS
Stallkamp et al. [31]	No	98.98		
Atif et al. [29]	No	100.00	99.80	99.31
Agrawal et al. [45]	No	* 77.43		
Youssef et al. [33]	No	95.00		98.20
Mathias et al. [1]	No		97.83	
Huang et al. [44]	No	* 95.56		
Lin et al. [51]	No		* 99.18	
Li et al. [28]	No	97.40	98.10	
Li and Wang [3]	No	99.66		
Zeng et al. [83]	No		95.40	
**Our Approach**	**No**	**100.00**	**99.72**	**96.03**
	**Yes**	**100.00**	**100.00**	**100.00**

Note: Accuracy values with * represent average accuracy across different classes of traffic signs no balanced accuracy was provided.
